# Management of granulomatous lobular mastitis: an international multidisciplinary consensus (2021 edition)

**DOI:** 10.1186/s40779-022-00380-5

**Published:** 2022-04-26

**Authors:** Qian-Qian Yuan, Shu-Xuan Xiao, Omar Farouk, Yu-Tang Du, Fereshte Sheybani, Qing Ting Tan, Sami Akbulut, Kenan Cetin, Afsaneh Alikhassi, Rami Jalal Yaghan, Irmak Durur-Subasi, Fatih Altintoprak, Tae Ik Eom, Fatih Alper, Mustafa Hasbahceci, David Martínez-Ramos, Pelin Seher Oztekin, Ava Kwong, Cedric W. Pluguez-Turull, Kirstyn E. Brownson, Shirish Chandanwale, Mehran Habibi, Liu-Yi Lan, Rui Zhou, Xian-Tao Zeng, Jiao Bai, Jun-Wen Bai, Qiong-Rong Chen, Xing Chen, Xiao-Ming Zha, Wen-Jie Dai, Zhi-Jun Dai, Qin-Yu Feng, Qing-Jun Gao, Run-Fang Gao, Bao-San Han, Jin-Xuan Hou, Wei Hou, Hai-Ying Liao, Hong Luo, Zheng-Ren Liu, Jing-Hua Lu, Bin Luo, Xiao-Peng Ma, Jun Qian, Jian-Yong Qin, Wei Wei, Gang Wei, Li-Ying Xu, Hui-Chao Xue, Hua-Wei Yang, Wei-Ge Yang, Chao-Jie Zhang, Fan Zhang, Guan-Xin Zhang, Shao-Kun Zhang, Shu-Qun Zhang, Ye-Qiang Zhang, Yue-Peng Zhang, Sheng-Chu Zhang, Dai-Wei Zhao, Xiang-Min Zheng, Le-Wei Zheng, Gao-Ran Xu, Wen-Bo Zhou, Gao-Song Wu

**Affiliations:** 1 Department of Thyroid and Breast Surgery, Zhongnan Hospital of Wuhan University, Wuhan, 430071 China; 2 Department of Pathology, University of Chicago Pritzker School of Medicine, Chicago, IL 60637 USA; 3 Department of Surgical Oncology and Breast Surgery, Oncology Center, Faculty of Medicine, Mansoura University, Mansoura, 35516 Egypt; 4 Department of Breast Surgery, Beijing University of Chinese Medicine, Beijing, 100700 China; 5 Department of Infectious Diseases and Tropical Medicine, Faculty of Medicine, Mashhad University of Medical Sciences, Mashhad, 9177899191 Iran; 6 Breast Department, KK Women’s and Children’s Hospital, 100 Bukit Timah Road, Singapore, 229899 Singapore; 7 Department of Surgery, Department of Public Health, Department of Biostatistics, Bioinformatics and Medical Informatics, Inonu University Faculty of Medicine, 44280 Malatya, Turkey; 8 Department of General Surgery, Faculty of Medicine, Çanakkale Onsekiz Mart University, 17020 Çanakkale, Turkey; 9 Department of Radiology, Cancer Institute, Imam Khomeini Hospital, Tehran University of Medical Sciences, Tehran, 1419733141 Iran; 10 Department of Surgery, College of Medicine and Medical Sciences, Arabian Gulf University-Bahrain, Manama, 26671 Bahrain; 11 Department of Radiology, International Faculty of Medicine, Istanbul Medipol University, 34810 Istanbul, Turkey; 12 Department of General Surgery, Faculty of Medicine, Sakarya University, 54050 Sakarya, Turkey; 13 Department of Surgery, HiU Clinic, 170, Gwongwang-ro, Paldal-gu, Suwon, 16488 Korea; 14 Department of Radiology, Faculty of Medicine, Ataturk University, 25240 Erzurum, Turkey; 15 Academic Support and Education Center, Hırkai Serif District, Kececi Cesmesi Str, Doktorlar Building, B/7, 34091 Istanbul, Turkey; 16 Department of General and Digestive Surgery, Hospital General Castellon, Avda Benicassim S/N, 12812004 Castellón, Spain; 17 Radiology Department, Ankara Training and Research Hospital, 305018 Ankara, Turkey; 18 Department of Surgery, The University of Hong Kong, China; The University of Hong Kong-Shenzhen Hospital, Shenzhen, 518053 China; 19 University of Miami Health System and Miller School of Medicine, 1475 NW 12th Avenue, Miami, FL 33136 USA; 20 Department of Surgery, University of Utah, Huntsman Cancer Institute, Salt Lake City, UT 84112 USA; 21 Department of Pathology, Dr D Y Patil Medical College Hospital and Research Centre, Pimpri, Pune, 603203 India; 22 Department of Surgery, Johns Hopkins Breast Center at Bayview Campus, 4940 Eastern Avenue, Rm. A-562, Baltimore, MD 21224 USA; 23 Center for Evidence-Based and Translational Medicine, Zhongnan Hospital of Wuhan University, Wuhan, 430071 China; 24 Department of Diagnostic Ultrasound, Zhongnan Hospital of Wuhan University, Wuhan, 430071 China; 25 Department of Surgery, Affiliated Hospital of Inner Mongolia Medical University, Hohhot, 010110 China; 26 Center for Pathology and Molecular Diagnostics, Wuhan University, Wuhan, 430071 China; 27 Department of General Surgery, Fujian Provincial Hospital, Fuzhou, 350001 China; 28 The First Affiliated Hospital with Nanjing Medical University, Nanjing, 210029 China; 29 Key Laboratory of Hepatosplenic Surgery and the First Department of General Surgery, First Affiliated Hospital of Harbin Medical University, Harbin, 150007 China; 30 Department of Breast Surgery, Zhejiang University School of Medicine First Affiliated Hospital, Hangzhou, 310003 China; 31 Department of General Surgery, The Affiliated Hospital of Guizhou Medical University, Guiyang, 550004 China; 32 Department of General Surgery, Shanxi Provincial People’s Hospital, Taiyuan, 030012 China; 33 Department of Breast Surgery, Xinhua Hospital Affiliated to Shanghai Jiaotong University School of Medicine, Shanghai, 200092 China; 34 Department of Cardiothoracic Surgery, Zaoyang People’s Hospital, Zaoyang, 441299 Hubei China; 35 Department of Thyroid and Breast Surgery, The Second Hospital of Hebei Medical University, Shijiazhuang, 050004 China; 36 Department of General Surgery, Guangshan County People’s Hospital, Guangshan County, Xinxiang, 465499 Henan China; 37 Department of Breast Surgery, First Affiliated Hospital of Nanchang University, Nanchang, 330006 China; 38 Chinese Academy of Sciences, Beijing, 100045 China; 39 Department of General Surgery, School of Clinical Medicine, Tsinghua University, Beijing Tsinghua Changgung Hospital, Beijing, 102218 China; 40 Department of Breast and Thyroid Surgery, The First Affiliated Hospital of University of Science and Technology of China, Anhui Provincial Hospital, Hefei, 230001 China; 41 Department of Thyroid Surgery, First Affiliated Hospital of Kunming Medical University, Kunming, 650032 China; 42 Department of Oncology, Liwan Central Hospital of Guangzhou, Guangzhou, 510150 China; 43 Department of Breast Surgery, Peking University Shenzhen Hospital, Shenzhen, 518036 Guangdong China; 44 Department of Computed Tomography, Zhongnan Hospital of Wuhan University, Wuhan, 430071 China; 45 Department of General Surgery, Xinxiang Medical University First Affiliated Hospital, Xinxiang, 453100 Henan China; 46 Department of Breast Surgery, Guangxi Medical University Cancer Hospital, Nanning, 530021 China; 47 Department of General Surgery, Zhongshan Hospital Fudan University, Shanghai, 200032 China; 48 Department of Breast and Thyroid Surgery, Hunan Provincial People’s Hospital/The First Affiliated Hospital of Hunan Normal University, Changsha, 410005 China; 49 Department of Breast and Thyroid Surgery, Chongqing General Hospital, University of Chinese Academy of Sciences, Chongqing, 400013 China; 50 Department of General Surgery, Qinghai Province People’s Hospital, Xining, 810007 China; 51 Department of Thyroid and Breast Surgery, Qingdao Women and Children’s Hospital, Qingdao, 266000 Shandong China; 52 Department of Oncology, Xi’an Jiaotong University Second Affiliated Hospital, Xi’an, 710004 China; 53 Department of Cardiothoracic Surgery, Zaoyang First People’s Hospital, Zaoyang, 441299 Hubei China; 54 Department of Thyroid and Breast Surgery, Yichang Central People’s Hospital, Yichang, 443003 Hubei China; 55 Department of Thyroid Surgery, The Second Affiliated Hospital, Guizhou Medical University, Kaili, 556000 Guizhou China; 56 Department of General Surgery, Shanghai Changzheng Hospital, Shanghai, 200003 China; 57 Department of Surgery, Dongfeng General Hospital Affiliated with Hubei Medical College, Shiyan, 442001 Hubei China

**Keywords:** Granulomatous mastitis, Granulomatous lobular mastitis, Idiopathic granulomatous mastitis, Diagnosis, Treatment

## Abstract

Granulomatous lobular mastitis (GLM) is a rare and chronic benign inflammatory disease of the breast. Difficulties exist in the management of GLM for many front-line surgeons and medical specialists who care for patients with inflammatory disorders of the breast. This consensus is summarized to establish evidence-based recommendations for the management of GLM. Literature was reviewed using PubMed from January 1, 1971 to July 31, 2020. Sixty-six international experienced multidisciplinary experts from 11 countries or regions were invited to review the evidence. Levels of evidence were determined using the American College of Physicians grading system, and recommendations were discussed until consensus. Experts discussed and concluded 30 recommendations on historical definitions, etiology and predisposing factors, diagnosis criteria, treatment, clinical stages, relapse and recurrence of GLM. GLM was recommended as a widely accepted definition. In addition, this consensus introduced a new clinical stages and management algorithm for GLM to provide individual treatment strategies. In conclusion, diagnosis of GLM depends on a combination of history, clinical manifestations, imaging examinations, laboratory examinations and pathology. The approach to treatment of GLM should be applied according to the different clinical stage of GLM. This evidence-based consensus would be valuable to assist front-line surgeons and medical specialists in the optimal management of GLM.

**Supplementary Information:**

The online version contains supplementary material available at 10.1186/s40779-022-00380-5.

## Introduction

Granulomatous lobular mastitis (GLM) is a rare, chronic benign inflammatory disease of the breast. Pathologically, GLM typically manifests as non-caseating granulomatous lesions with leukomonocytes, lymphocytes, neutrophils and multinucleated giant cells, located in the center of breast lobules. With a rapidly increasing morbidity in the last two decades, GLM tends to occur in child-bearing women with a prolonged and recurrent course. Although clinical findings and histopathological features are necessary in the diagnosis of GLM, currently there are no international unified guidelines for GLM diagnosis and treatment. Difficulties may exist in the management of GLM for a significant number of front-line surgeons and medical specialists who care for patients with inflammatory disorders of the breast. To promote the standardization process of the diagnosis and treatment of GLM, 66 international experienced multidisciplinary experts from 11 countries or regions proposed the guideline about diagnostic strategy and management algorithm of GLM based on published literature. This guideline statement aims to standardize diagnostic approach, differential diagnoses and clinical management strategies which can be applied to all medical institutions managing GLM patients.

## Methods

### Literature evidence

Search parameters for the literature were set from January 1, 1971 to July 31, 2020. This time frame was extended back to allow for inclusion of the first published literature of GLM. For each topic, the primary coauthor conducted a search in PubMed using the Medical Subject Headings and Boolean operators for “granulomatous lobular mastitis”, “granulomatous mastitis”, “idiopathic granulomatous mastitis” search terms. References included language in English and several in Chinese. The literature search retrieved a total of 509 articles from PubMed.

### Grading of practice recommendations

The 2010 American College of Physicians (ACP) grading system, which employs a validated scale to critically interpret and evaluate the strength and quality of the evidence and provide guidance on how to best apply the recommendations to individual patients, was utilized in manuscript preparation [1]. The ACP system applies the terms “strong” when benefits clearly outweigh risks and/or the recommendation should be applied to all or most patients without reservation, ‘‘weak’’ when benefits are finely balanced with risks or appreciable uncertainty exists. The quality of the evidence was graded “high” for well-done randomized controlled trials or overwhelming evidence, “moderate” for randomized controlled trials with important limitations, well-designed cohort or case–control studies, or large observational studies, and “low” for potentially biased, small observational, or case studies. When the evidence is insufficient to determine for or against routinely provided service, the authors grade the recommendation as “insufficient evidence to determine net benefits or risks”.

## Historical perspective and definitions

***Recommendation 1: The consensus experts group recommends GLM as a widely accepted definition. (Strong recommendation, high quality of evidence)***

Comments: In 1971, Miller et al. [2] proposed the concept of granulomatous mastitis (GM) which presents as breast lobules infiltrated with acute and chronic inflammatory exudate with a mass of foreign body giant cell. In 1972, Kessler and Wolloch [3] described the characteristics of GM: child-bearing women, 1.5–5 years since the last delivery, multiple granulomas and abscess formation. In 1987, Going et al. [4] emphasized the histologic characteristics (the lesions located in the center of breast lobule) and recommended replacing GM with GLM. In 1994, Donn et al. [5] began to apply the definition of idiopathic granulomatous mastitis (IGM) to emphasize the unclear etiology. In 2010, Boarki and Labib [6] proposed idiopathic granulomatous lobular mastitis (IGLM) based on the etiological and histologic diagnosis. In 2011, Renshaw et al. [7] described a special histologic category, vacuole-like cavity formed by neutrophils, and Gram-positive bacilli could be detected in the cavity concomitant with diffuse granuloma, which was defined as cystic neutrophilic granulomatous mastitis (CNGM). Some researchers considered CNGM as a subtype of GLM [8, 9], and others classified CNGM as GM [10–12].

As understanding of this disease becomes more comprehensive, related terms and definitions continue to evolve, bringing confusion to clinicians. GLM is a histopathological diagnosis while IGM is an etiologic diagnosis. GLM has been found to be closely related with *Corynebacterium* infection [10, 13]. However, whether the detection of *Corynebacterium* is positive or negative, the same pathological characteristics are shown in specimens with GLM. Therefore, GLM avoids the problem about undetermined etiological relationship between *Corynebacterium* infection and disease development.

Even though GLM and GM are both pathological terms describing inflammatory changes in breast, GM may be further divided into primary (idiopathic) and secondary (infectious and non-infectious): (1) Primary (idiopathic) granulomatous mastitis: An underlying cause cannot be detected by routine pathogenic examination. Idiopathic granulomatous mastitis is a diagnosis that has excluded other forms of granulomatous mastitis with definite etiologies. (2) Secondary granulomatous mastitis: 1) infectious granulomatous mastitis: pathogenic microorganism can be detected by etiological examination, such as tuberculosis, actinomycetes, parasites, fungi and histoplasma, etc.; 2) non-infectious granulomatous mastitis: etiology is clear while no pathogen can be detected. Owing to the breast involvement by autoimmune disease or immune rejection response to exogenous material, non-infectious granulomatous mastitis tends to be a benign breast disease concomitant with granulomatous changes. This includes Wegener's granulomatous mastitis, giant cell arteritis mastitis, sclerosing lymphogranulomatous inflammation of the breast, foreign body granulomatosis, sarcoidosis and fat necrosis, etc..

## Etiology and predisposing factors of GLM

### Predisposing factors of GLM

***Recommendation 2: Lactation disorders resulting in milk stasis, hyperprolactinemia, and blunt trauma of breast are the predisposing factors of GLM. (Strong recommendation, moderate quality of evidence)***

***Recommendation 3: The prevalence of GLM is associated with race and region. (Strong recommendation, moderate quality of evidence)***

Comments: Possible predisposing factors of GLM include lactation disorders that result in milk stasis, hyperprolactinemia, and blunt trauma of breast [14–17]. Milk stasis plays a key role with breast tissue developing into a hypertrophic state subsequent to pregnancy, lactation, and hyperprolactinemia. Pituitary adenoma, antipsychotic drugs (such as potent D2 receptor antagonists, risperidone), and antidepressant drugs (such as selective serotonin reuptake inhibitors, fluoxetine) can lead to hyperprolactinemia [18, 19]. While the permeability of breast ducts increases, the immunogenic substance (retained milk) enters into lobular mesenchyme of the breast, causing T cell-mediated immune response and granuloma formation [20]. It is not clear in the literature how oral contraceptives are predisposing factors for GLM [21]. The nature of extramammary manifestations of GLM, including inflammatory arthritis, arthralgias, episcleritis, and erythema nodosum, is suggestive of an underlying immune process [22]. A favorable response to corticosteroids is supportive of this pathogenesis [22]. However, serological tests that are usually positive in patients with autoimmune diseases, such as rheumatoid factor and antinuclear antibodies have not shown consistent results with diagnostic or prognostic value [23].

Differences in race and region exist in the prevalence of GLM. GLM may be associated with dietary habits and genetic factors [14, 21]. As GLM usually occurs in the Mediterranean region and developing Asian countries. This prevalence might be the reflection of under-diagnosis of tuberculosis mastitis. Sometimes routine histology studies are not sufficient to rule out the diagnosis of tuberculosis mastitis. Presence of atypical mycobacteria may also be involved in the pathogenesis of GLM which are difficult to isolate under routine culture conditions. This may also be related to poor habits of lactation and weaning in Mediterranean region that may lead to milk stasis (galactostasis) which is the most important predisposing factor for GLM. [(1) Some GLM patients depend on lactation from one breast only and neglect lactation from the other one which develop GLM due to milk stasis. (2) Many patients neglect the routine breast massage and complete evacuation of both breasts after each time of lactation. (3) Many patients also neglect the routine breast massage and complete evacuation of both breasts during the first weeks of weaning.]

### Pathogenesis of GLM

The most widely adopted theory considers GLM to be an immune reaction that involves both humoral and cell-mediated immunity stimulated by patients’ secretions such as retained milk [22, 24]. Deng et al. [25] retrospectively investigated steroids administration after the vacuum-assisted biopsy (VAB) of GLM, and immunohistochemical (IHC) staining for immune-related antigens (CD3, CD4, CD8, CD79a, IgG, and IgM) was performed. The results revealed that CD3, CD4 and CD8 lymphocytes were present diffusely in the lesion, indicating that cell-mediated immunity is involved in the development of GLM, and CD79a lymphocyte positivity indicates that humoral immunity is involved [25]. Kessler et al. [3] first reported the self-limited course of GLM, Cohen [26] also considered GLM as focal lesions of autoimmune disease. Available evidence on the effectiveness of glucocorticoids and immunosuppressive therapy for treatment of GLM supports this hypothesis [27].

**Fig. 1:**
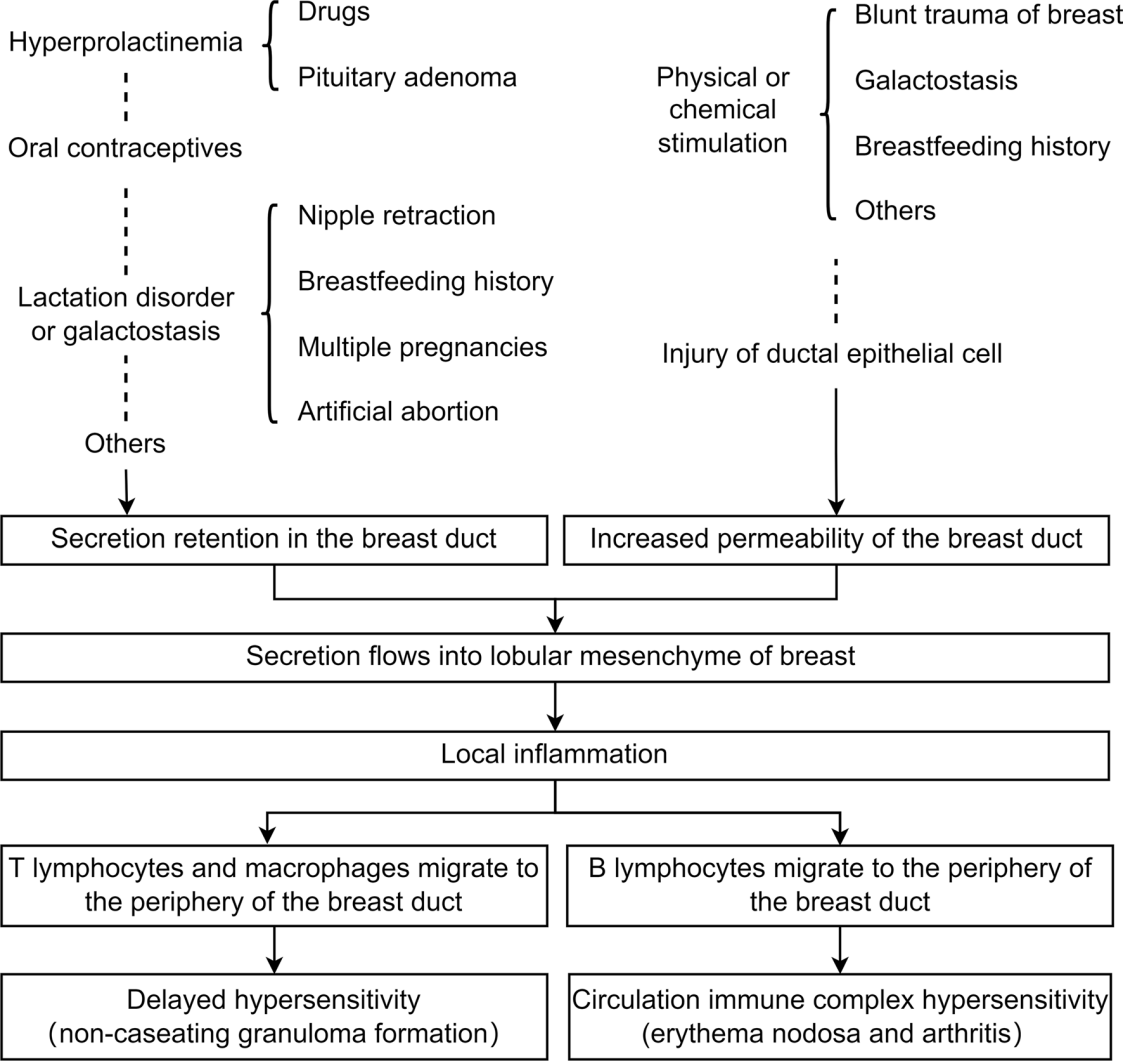
Pathogenesis of granulomatous lobular mastitis (GLM)

The pathogenesis of GLM may be due to increased permeability of breast duct caused by physical or chemical stimulation such as the infiltration of the lobular mesenchyme of breast with intraluminal secretions such as retained milk. This causes local inflammation in the mesenchyme which then induces the infiltration of immunocompetent cells to form delayed-type hypersensitivity. Finally, localized granulomas are formed (Fig. 1) [3, 28, 29].

### Relationship between GLM and *Corynebacterium* infection

The relationship between GLM and *Corynebacterium* infection is not yet definitive. In 2003, Taylor et al. [30] first detected *Corynebacterium* in lesions of GM, of which *Corynebacterium kroppenstedtii* accounted for 14.1%. However, recent literature indicates a potential causal relation between CNGM and *Corynebacterium.* CNGM is a recently identified as an entity that raised the reconsideration of an underlying bacterial etiology for GLM [10, 12, 23, 31]. Typical biopsies of CNGM reveal a granulomatous inflammation with characteristic cystic spaces lined by neutrophils containing Gram-positive cocci [8]. *Corynebacteria*, especially *Corynebacterium kroppenstedtii*, and to a lesser extent *Staphylococcal spp*. were detected in some patients with CNGM by culture, or by 16S rRNA gene sequencing of specimens obtained at surgery or FNAC [8, 10]. Kivilcim et al. [32] evaluated multiple bacteriologic agents that might play a role in the etiology of GLM using a nucleic-acid-based assay with a universal primer on previously obtained GLM biopsies without evidence of CNGM [8]. They obtained no positive results [8]. The minority of cases diagnosed in the past as GLM with evidence of *Corynebacterium* may turn to be CNGM. In brief, when the characteristic histological findings of CNGM are encountered, the pathologist should make every effort to look for an associated primary bacterial infection. Culture of fresh specimens, Gram staining, or rRNA gene sequencing of specimens obtained at surgery might be considered in these cases [10, 31]. CNGM is now considered a secondary form of GM [10, 12, 23]. Early recognition of CNGM might call for a first line of treatment by lipophilic antimicrobials [12].

The normal flora in the breast is consistent with the skin commensal flora, mainly *Propionibacteria*, coagulase-negative *Staphylococci* and *Corynebacteria*, which can enter tissue through mammary ducts. Nipple retraction may be a predisposing factor leading to bacterial retrograde infection. Patients with *Corynebacterium* infection are more likely to present with fever and sinus formation [8]. If *Corynebacteria* are the causative factor in the development of GLM, the latter should be classified as infectious granulomatous mastitis. However, as a part of normal skin flora, the presence of *Corynebacterium* may be a colonization. Therefore, there are three hypotheses to explain the pathogenetic of GLM in relation with *Corynebacterium*. (1) Primary factor: As an independent immunogenic factor, *Corynebacterium* enters lobular mesenchyme of breast through increased permeability of breast ducts, inducing an autoimmune response. (2) Cofactor: *Corynebacterium* and immunogenic factors such as retained milk enter the lobular mesenchyme through increased permeability of breast ducts, and induce an autoimmune response. (3) Irrelevant factor: *Corynebacteria* does not participate in the process of autoimmune response. Other immunogenic factors acting as antigens enter lobular mesenchyme through increased permeability of breast ducts. Decreased local immune resistance and secondary *Corynebacterium* infection then exacerbate clinical symptoms.

### Odds in the etiology

Despite a universal agreement on the relation of GLM to parity and lactation, possibly reflecting the impact of prolonged estrogen exposure, still it is rarely reported in nulliparous women, young girls, and males, expanding the dilemma about the etiology [22, 23, 33]. It is worth mentioning, however, that in a good percentage of cases reported in the male population, there was suggestion of increased endogenous estrogen to androgen ratio [33].

## Diagnosis criteria

***Recommendation 4: The diagnosis of GLM requires cooperation through the establishment of an effective multidisciplinary comprehensive treatment group, including clinicians, radiologists and pathologists. (Strong recommendation, moderate quality of evidence)***

***Recommendation 5: The diagnosis of GLM should combine history characteristics, clinical manifestations, physical examination, imaging examination and laboratory examination. (Strong recommendation, moderate quality of evidence)***

Comments: There is evidence that the establishment of a multidisciplinary team for the management of GLM reduces rate of erroneous initial clinical impression of breast cancer [23]. Such a multidisciplinary approach achieved a higher rate of earlier preoperative diagnosis of GLM expanding the role of corticosteroid treatment in selected patients. False positive radiological diagnosis of breast carcinoma was also reduced [23].

### Clinical history characteristics

***Recommendation 6: GLM usually occurs in child-bearing women, seldom in pregnant and lactating women and very rarely in men. (Strong recommendation, high quality of evidence)***

***Recommendation 7: GLM in patients who have not been pregnant or delivered is associated with increased serum prolactin, or up-regulated prolactin receptors or hypersensitivity to the normally circulating prolactin. (Strong recommendation, high quality of evidence)***

Comments: Clinical history information include age, body mass index (BMI), course of disease, history of child-bearing (number of pregnancies and deliveries, date of last delivery), history of lactation (episodes of acute mastitis during lactation, date of last breastfeeding, problems during breastfeeding, lactation from one breast only and negligence of lactation from the other one, negligence the routine breast massage and complete evacuation of both breasts after each time of lactation and during the first weeks of weaning), history of blunt trauma to the breast, history of taking oral contraceptives, history of taking psychiatric drugs, historical symptoms or history of autoimmune disease.

A descriptive study had found that median age for patients with GLM is 36 years (19–49 years) [34]. Azizi et al. [35] reported that patients with GLM usually had history of pregnancy (90.7%) and history of breastfeeding (82.7%). In the prospective study of Farouk et al. [21] that included 30 patients diagnosed with GLM, all patients were of reproductive age, and all had a history of breast feeding (100%). Male GLM had been documented in 13 cases, with a suggestion of higher endogenous estrogen to androgen ratio in a few of them [33]. Some case reports described GLM associated with hyperprolactinemia, which might result from specific drugs or pituitary adenoma, including antipsychotic drugs such as risperidone and antidepressant drugs such as fluoxetine [18, 36].

### Clinical manifestations

***Recommendation 8: In the early stage, breast mass can arise with or without pain, and without obvious local skin changes. The lesions usually spread to the areola from the periphery of the breast. (Strong recommendation, high quality of evidence)***

***Recommendation 9: Breast masses can enlarge rapidly with inflammatory manifestations including hyperaemia, swelling, warmth, and pain. Systemic inflammatory findings like fever usually does not occur. (Strong recommendation, high quality of evidence)***

***Recommendation 10: In a small number of patients with GLM, focal mastitis or tenderness may arise before the development of a mass by 2 to 3 months. (Strong recommendation, moderate quality of evidence)***

***Recommendation 11: During the progression of GLM, deep or subcutaneous abscesses can form. (Strong recommendation, high quality of evidence)***

***Recommendation 12: In the advanced stage, the main clinical manifestation is a fistula or sinus formation. The skin can be ulcerated and discharge purulent with prolonged healing time. (Strong recommendation, high quality of evidence)***

***Recommendation 13: Systemic involvement such as erythema nodosum and oligo- or poly-arthritis can appear at any stage and can be in favor of the diagnosis of GLM.***

Comments: Clinical manifestations of GLM are diverse. A descriptive study reported 3060 patients with GLM, of which breast mass was the most frequent symptom (80%) with a mean size of 5 cm (3 – 9 cm), and 66% of them were painful [34]. Azizi et al. [35] described clinical symptoms of 474 GLM patients, 39.2% had skin lesions, 17.7% had concomitant nipple retraction, 15.6% had nipple discharge, and 4.6% had joint pain. Co et al. [37] reported 55.9% (57) of 102 GLM patients presenting with a painful mass, 28.4% presented with a painless mass and 15.7% had abscess formation. The median size of the inflammatory mass or abscess was 37 (6–92) mm.

The first ten reported cases of GLM by Kessler and Wolloch [3], and Cohen [26] shared the classical presentation of a hard cancer-mimicking breast mass occurring in young parous women. However, the spectrum of possible local presentations GLM has expanded to include acute breast-abscess-like presentations, and the subacute presentations with skin fungation, fistulization, and/or sinus formation [21]. Extramammary presentations in the form of erythema nodosum, arthritis, oligo-arthritis, and episcleritis are recognized as occasional findings [21, 22, 33, 38].

### Imaging examinations

***Recommendation 14: Ultrasound (US) is the first line modality for GLM. US helps to detect the inflammatory changes, abscess, and tunneling and sinus formation; US is also helpful to perform biopsy and do follow-up of remission or progression of GLM. (Strong recommendation, high quality of evidence)***

***Recommendation 15: Magnetic resonance imaging (MRI) is a useful imaging modality for the differential diagnosis with breast cancer and it also could be useful to indicate active lesions, locate the extent of the lesions, evaluate possible residual disease after treatment or monitor the disease in patients who underwent conservative treatment. (Strong recommendation, moderate quality of evidence)***

***Recommendation 16: Hyperprolactinaemia or suspected TB should be appropriately investigated. (Strong recommendation, moderate quality of evidence)***

Comments: Irregular hypoechoic masses, tubular echoes, and multiple abscesses can be observed by US [39]. When the lesion is fibrous or blood flow of the lesion is rich, obvious acoustic shadow can be observed behind the lesion during US examination. In the absence of a mass lesion, US evidence of areas of mixed echo pattern with parenchymal deformity, multiple collection pockets and tracks, focal mastitis with interstitial edema, may indicate the presence of an inflammatory granulomatous process [40]. Alikhassi et al. [41] analyzed imaging features in 36 patients with GLM, 72.2% of them presented irregular, less uniform, hypoechoic masses with ill-defined margin, 50% of them formed tubular dilation and subcutaneous sinus formation, 28% of them showed floating debris, and 25% of them existed ductal ectasia.

In mammography, focal or global asymmetric density, blurred edges, with or without skin thickening and parenchymal distortion can be detected in GLM patients [42]. Aghajanzadeh et al. [43] reported that the major mammographic finding was an irregular mass in 118 (63.5%) of 186 GLM patients, asymmetric density and heterogeneously were found in 8.5% patients, 5% of them presented skin thickening or edema, and 3.5% of them showed an irregular or lobulated mass. Young child-bearing women who tend to develop GLM, have dense breast parenchyma is dense, making the detection of lesion difficult. Thus GLM could be misdiagnosed as breast cancer [44].

The characteristics of GLM detected by MRI commonly present as heterogeneous enhancing masses, segmental non-mass enhancement, or focal non-massive lesions [45]. MRI shows edema, inflammation, tumor-like lesions, as well as abscess fistula formation in the parenchyma [46]. Micro-lesions with fusion, T_2_ high signal intensity lesions and rim enhancing micro-abscesses are among other MRI presentations [47]. Most lesions in GLM show persistent dynamic curves, but the enhancement curves of different parts of a lesion in a patient may not be the same [48]. Decreased ADC sequence signal is observed in GLM on diffusion-weighted imaging (DWI), which is of little value in distinguishing from inflammatory breast cancer [49]. Zhang et al. [50] revealed that the accuracy of MRI-enhanced imaging in assessing the extent of GLM lesions was 88.9% (24/27), much higher than the accuracy of US alone or combined with mammography. Furthermore, MRI can provide more accurate information regarding the assessment of therapy success especially after the local therapy [51, 52]. MRI is a follow-up tool for aggressive, diffuse, and non-responsive diseases [53].

### Laboratory examination

***Recommendation 17: Laboratory examinations include routine blood test, erythrocyte sedimentation rate, purified protein derivative (PPD) test and inflammatory markers such as C-reactive protein, serum prolactin, and immunological examination such as antinuclear antibody (ANA) profile and rheumatism factor. (Strong recommendation, high quality of evidence)***

***Recommendation 18: Testing tissue for the presence or growth of Mycobacterium tuberculosis should be performed for patients with suspected tuberculosis. (Strong recommendation, moderate quality of evidence)***

Comments: PPD of tuberculin test can be useful in weighing the differential diagnosis. There is a clinical anergic response to PPD among GLM patients that could be useful in ruling out TB and for diagnosis of latent TB while deciding to prescribe immunosuppressant medication to GLM patients [54]. Inflammatory cells increase in GLM patients. For GLM patients with erythema nodosa and arthritis, C-reactive protein abnormally increases, and ANA and rheumatoid are normal [55]. DNA test of Mycobacterium tuberculosis helps to distinguish tuberculous granulomatous mastitis [56]. Patients who have not been pregnant or delivered often suffer from hyperprolactinemia or up-regulated prolactin receptors or hypersensitivity to the normally circulating prolactin [57].

### Histopathology

***Recommendation 19: Core needle biopsy (CNB) can be performed as diagnostic histopathological examination (strong recommendation, high quality of evidence)***

Comments: GLM is characterized histologically as non-caseating granulomatous lesions with epithelioid histiocytes and multinucleated giant cells, located in the center of the lobules (Additional file 1: Fig. S1). The surrounding tissue is mainly infiltrated by neutrophils, lymphocytes, plasma cells and a small number of eosinophils. The lesions can be multifocal and form micro-abscesses, and vary in size. Inflammation of GLM is usually confined to the breast lobules, seldomly involving the main ducts [58, 59]. The gross pathological appearance of GLM is nonspecific, and depends on the size of the lesion, degree of inflammation, fibrosis, and micro-abscess formation.

Routine biopsy methods include CNB, VAB, and fine needle aspiration (FNA); the former two methods have a high accuracy for diagnosis. Excisional biopsy can be performed if necessary. Frozen section evaluation can occasionally be useful in confirming the diagnosis and judging the extent of surgical resection [23]. A study by Hovanessian Larsen et al. [44] indicated that 21% (4/19) of GLM cases can be diagnosed by FNA, and 96% of GLM patients can be diagnosed by CNB. In addition, it is necessary to pay attention to the site selection of CNB, inserting the needle as close as possible to the margin of the areola. If a subsequent operation is planned, the puncture tunnel can be removed at the same time to reduce scars.

### Differential diagnosis

GLM needs to be differentiated from ductal dilatation/periductal inflammation of breast, Zuska disease/subareolar abscess, and breast cancer (Table 1).

**Table 1:** Differential diagnosis of GLM

	Etiology	Age	Clinical manifestations	Auxiliary examination	Histopathology
GLM	Unknown etiology. Motivations are blunt breast trauma, lactation disorder, galactostasis, hyperprolactinemia, etc. [60]	Women who have delivered with breastfeeding history tend to develop GLM. Women who have not delivered seldom develop GLM	It frequently occurs on the periphery of the breast and concentrically involves the areola area. The subcutaneous abscess can spread to the whole breast, and can form recurrent ulcers or sinuses with a prolonged healing time	Ultrasonography often presents hypoechoic or uneven masses, with or without duct dilation	Non-caseating granulomas centered on the breast lobular, distributed multifocally, varying in size, with or without micro-abscesses
Ductal dilatation/periductal inflammation of the breast	Unknown etiology. Ductal dilatation may be associated with nipple deformities, blocked milk ducts, smoking, and bacterial infections [61]	Women in all age groups can develop ductal dilatation, more often in perimenopausal women. Women who have not been delivered can develop ductal dilatation as well	Manifestations include nipple discharge with nipple retraction. The lesion is centered on the areola [62], showing eccentric development. The large ducts behind the areola dilate, and an areola abscess may appear	Obvious duct dilation, fine light spots inside, and flow signs when pressurized can be observed by ultrasound. Dilated, tortuous, blocked and deformed duct can be observed by galactography	Breast duct is highly dilated, the wall of the duct is thickened or ruptured, and the cyst cavity is filled with pink granular thick material. Infiltration of lymphocytes, plasma cells and neutrophils can be seen around the dilated duct [63]
Zuska disease/subareolar abscess	Zuska may be associated with the breast duct obstruction, congenital malformation of breast duct, and nipple retraction	Zuska mainly occurs in non-lactating period, more common in women aged 14–66 years old, especially unmarried women	Swelling under the areola, swelling formation or abscess, lactiferous fistula and repeated attacks, prolonged non-healing are important characteristics of this disease	One or more hypoechoic or anechoic areas with blood flow signals can be detected around the areola by ultrasound	Squamous metaplasia of lactiferous tube columnar epithelium at the base of the nipple
Breast cancer	Breast cancer may be related with a family history of breast cancer, BRCA1/2 mutation [64], exposure of radiation, first menstruation before 12 years old, first pregnancy after 35 years old, no pregnancy, tobacco and alcohol, and psychological stress. Oncogenic and latent viruses such as HPV, CMV, EBV, MMTV and BLV are recently accused to be etiologic factors in the pathogenesis of breast cancer. Moreover, stress has a role in activation of these viral mechanisms	The incidence rate gradually rises after the age of 20, and more frequently in perimenopausal and post-menopausal women	Breast cancer usually presents a single mass with unclear borders, hard texture and poor mobility, and may be accompanied by enlarged ipsilateral axillary lymph nodes. As a rare subtype of breast cancer, inflammatory breast cancer develops rapidly, and local skin may show inflammation-like manifestations, including redness, edema, thickening, roughness, and increased surface temperature [65]	A hypoechoic mass with unclear borders and blood flow signals can be detected by Doppler ultrasound[66]. Ultrasound manifestations of inflammatory breast cancer include thickened skin and extensive parenchymal echo enhancement of the breast. Mammography shows an increased density of masses with irregularity margin or with Burr sign, small and dense calcification	**–**

*BLV* bovine leukemia virus, *CMV* cytomegalovirus, *EBV* Epstein–Barr virus, *GLM* granulomatous lobular mastitis, *HPV* human papillomavirus, *MMTV* mice mammary tumor virus

## Treatment

The treatment strategy should be the responsibility of a multidisciplinary team. Individual patient needs should be taken into consideration.

### Etiologic treatment

***Recommendation 20: GLM patients with hyperprolactinemia should be treated with bromocriptine and the etiology should be cured. For patients with hyperprolactinemia caused by antipsychotic drugs such as risperidone, drug substitution should be evaluated by the physician and psychiatrist. (Strong recommendation, high quality of evidence)***

Comments: Hyperprolactinemia or up-regulated prolactin receptors or hypersensitivity to the normally circulating prolactin may be involved in the pathogenesis of GLM [67]. GLM patients with hyperprolactinemia have a higher risk of recurrence [68]. Nikolaev et al. [36] proposed that the usage of low-dose corticosteroids is effective in patients with hyperprolactinemia who have had pituitary adenoma resected. Aghajanzadeh et al. [43] revealed that the combination of glucocorticosteroid and bromocriptine (5–10 mg/day) was effective in 31% (5/16) GLM patients.

### Clinical and sonographic follow-up

***Recommendation 21: As a self-limiting disease, the symptoms of GLM can be relieved without any treatment. If small breast masses are the only symptoms, without other systemic symptoms, patients can be supervised after identifying the etiology of the mass. The progression of GLM needs to be closely monitored. (Weak recommendation, low quality of evidence)***

Comments: 50% of the GLM patients would achieve complete remission at 2–24 months since disease onset, the other 50% have no progression in the disease [69]. Davis et al. [70] surveilled 120 GLM patients from 2006 to 2019, 112 of them achieved complete remission on average 5 months (0–20 months). Hur et al. [71] managed 50 GLM patients on grade according to the severity of symptoms. Eight patients with milder illness were supervised, 5 of them had single/multiple small lesions (1–2 cm). Seven patients achieved remission, and one patient with large mass (5 cm) developed into an abscess.

### Medical treatment

Usually, drug acts as the first-line primary treatment or secondary treatment pre- and post-operation.

#### Antibiotics

***Recommendation 22: Antibiotics can be applied based on the results of bacterial testing and drug susceptibility tests. (Strong recommendation, moderate quality of evidence)***

Comments: GLM patients with *Corynebacterium* infection require antibiotics [72]. Dobinson et al. [13] conducted a drug susceptibility analysis for 27 *Corynebacteria* infected samples from breast, showing that *Corynebacterium kluyveri* was resistant to β-lactam antibiotics. Non-lipophilic *Corynebacterium* such as *Corynebacterium glucoside*, and *Corynebacterium frenii* are sensitive to multiple antibacterial drugs. For patients suspected of GLM, non-penicillin drugs such as clindamycin [73], levofloxacin and azithromycin can be applied empirically before the outcome of antibiotic susceptibility test [72, 73].

Mixed infective micro-organisms may be involved in the pathogenesis of GLM and some of these are atypical microorganisms, which are difficult to isolate under ordinary culture conditions. Rifampicin inhibits the growth of most Gram-positive and many Gram-negative bacteria, including atypical *Mycobacteria* that may be involved in the pathogenesis of GLM. Therefore, the use of Rifampicin can be used for GLM patients with *Corynebacterium* infection in all stages and may be applied for GLM patients without *Corynebacterium* infection as well [21]. In the prospective study that was included 30 patients diagnosed with GLM, Farouk et al. [21] have successfully proven the efficacy of a Rifampicin therapy regimen of 300 mg twice daily for a period of 6–9 months in the treatment of GLM for all patients at all stages with complete clinical and ultrasonographic response without any recurrent episodes after a median follow-up of 15.5 months (average 3–35 months) without any surgical excision or corticosteroid therapy.

#### Corticosteroids

***Recommendation 23: Administration of corticosteroids for large lesions prior to surgery may help in obtaining better cosmesis. Treatment time needs to be adjusted according to the progression of disease. (Strong recommendation, high quality of evidence)***

***Recommendation 24: GLM patients with mainly skin changes or who suffered from side effects of oral corticosteroids can be treated with intralesional corticosteroid injection and topical steroid. (Weak recommendation, moderate quality of evidence)***

Comments: A growing number of publications over the last two decades have shown the effectiveness of oral corticosteroid treatment in reducing the extent of surgery, or even alleviating the need for surgery in selected cases. On the other hand, the use of corticosteroids might be limited in pregnant, diabetic, or lactating women. The usage of glucocorticosteroid for a prolonged time may lead to weight gain, osteoporosis, and worsening infections [22, 33]. Although there were many studies considering the dosage, time, and methods to apply corticosteroids, there were no studies regarding the end-point to discontinue the treatment up to date. Complete clinical response (CCR) was the criteria to discontinue the treatment in the search of the effectiveness of steroid treatment, in accordance with the studies to date [24]. The usage of glucocorticosteroid should comply with the principle of minimum effective dose. However, Montazer et al. [74] had reported that high dose prednisolone has a high success rate with lower recurrence and could reduce the need for surgery. Çetin et al. [75] found the systemic side effects could not reduce significantly with the decreased dose of systemic steroids in combination with topical treatment.

Intralesional injection and topical corticosteroids can effectively reduce the side effects, especially in patients suffering from concomitant skin lesions (e.g., fistula, skin erosions, ulcers) [46, 75, 76]. For breast masses with single or multiple abscesses or even sinus, Xiao et al. [77] recommended aspiring pus repeatedly, guided by US, wash the abscess cavity using 0.9% NaCl solution, followed by injecting 40 mg triamcinolone acetonide into the abscess cavity through the aspiration needle or drainage tube. The therapeutic efficiency was 78.26% (18/23), and the effective time is (6.00 ± 2.09) d [77]. Tae Ik Eom recommended 6–7 times of triamcinolone acetonide injection (maximial dose 20 mg with interval every 3 weeks). Furthermore, intra-mammary corticosteroids injection can also be administered to perilesional fibroglandular area in patients without abscess formation. The major advantage of this method is that it can be applied in multiple sessions until satisfactory results were obtained [78]. Toktas et al. [79] divided 78 female patients diagnosed with GLM into the local steroid treatment group (intralesional steroid injection with topical steroid administration, group 1) and the peroral systemic steroid treatment group (group 2). The recurrence rates were significantly lower in group 1 (8.7%) compared to group 2 (46.9%, *P* = 0.001), and the need for surgical treatment was significantly less in group 1 (2.2%) than in group 2 (9.4%, *P* = 0.001), while the complication rates were similar between groups.

#### Non-corticosteroid immunosuppressive agents

***Recommendation 25: For patients who are resistant to corticosteroids or intolerant of long-term corticosteroids therapy, non-corticosteroid immunosuppressive agents such as methotrexate (MTX) may be considered. (Weak recommendation, low quality of evidence)***

Comments: The combination of MTX and corticosteroids has a synergistic effect on the control of disease progression, meanwhile the dosage of corticosteroids can be reduced [27, 80, 81]. The dosage of MTX is 5–15 mg/week for 6–24 months, and patients receiving MTX should be given two doses of folic acid per week before MTX use [82]. For women of reproductive age receiving MTX, contraception should be provided. Side effects should be closely observed during treatment, such as impairment of liver and renal function, bone marrow suppression, interstitial pneumonia, folic acid deficiency, and gastrointestinal reactions [54, 83]. Azidothymidine (AZT) could be an option for pregnant women [84].

### Surgery

***Recommendation 26: Surgical treatment (wide local excision) is most effective in complex lesions with limited focus, sinus tract, and without abscess, including abscess excision and drainage, segmental dissection, enlarged dissection and mastectomy of breast. (Strong recommendation, high quality of evidence)***

Comments: Indications for surgical operations: (1) When patients are not sensitive to the corticosteroids and antibiotic therapy, or cannot tolerate the side effects of corticosteroids. (2) Patients who recur after corticosteroid or surgical treatment. (3) The lesions are extensively distributed in three quadrants of the breast. (4) Complicated lesions with acute and chronic manifestations such as abscesses, sinus, fistula formation, and persistent wound infection (skin ulcers and pus). (5) Patients with long course of disease combined with systemic manifestations such as erythema nodosa and polyarthritis of extremities.

Relative contraindications for surgery: (1) Patients with symptoms of acute infection or are in the advanced stage of disease. (2) Extensive lesions with involving more 2/3 of the breast, wide area of skin lesions, difficulties in guaranteeing a satisfactory recovery after surgery. (3) Pregnant patients.

Surgical approaches: There is no unified surgical methods for GLM, which mainly depend on the surgical extent and lesion location. Wide local excision has stood the test of time as being a corner stone in the treatment of GLM. First, locate lesions preoperatively by US or MRI to identify the surgical extent to fully remove the necrotic tissue and pus. Then, completely wash the wound with 3% hydrogen peroxide, iodine and 0.9% saline. Finally, breast plastic surgery can be performed with the glandular fascia flap and fascia tissue. Immediate breast reconstruction can be conducted if necessary.

Notifications: (1) The selection of operative incision should consider both areola and ulcer. (2) Changing instruments and gloves to avoid re-contamination during the reconstruction surgery. (3) Double-layer purse-string suture at the base of the nipple makes the nipple protrude to avoid postoperative indentation. (4) For patients with extensive local resection, autologous tissue displacement and shaping can be performed.

Summary of evidence: In a recent systematic review and meta-analysis surgical treatment (with or without corticosteroids) was associated with a high cure rate and a relatively low recurrence rate, the cure rates of oral corticosteroids and surgery were 90.6%, 94.5%, respectively, and the recurrence rates were 6.8%, 4.0%, respectively [24]. For patients with diffuse disease, recurrence, or ineffective conservative treatment, wide local excision can be applied.

## Clinical management pathways

**Fig. 2:**
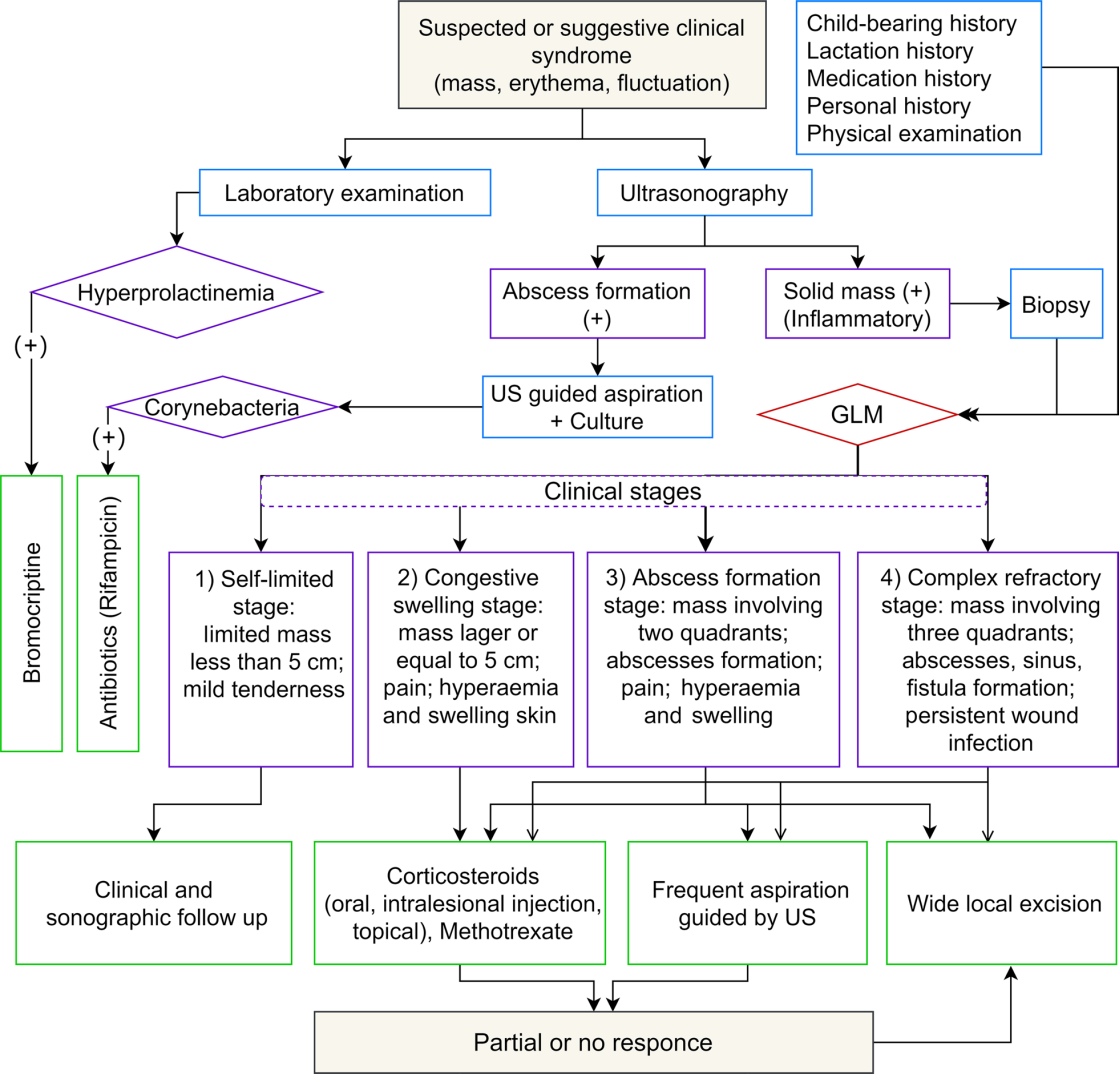
Clinical management algorithm of GLM. US ultrasound, GLM granulomatous lobular mastitis

GLM is classified into four stages according to the progression of the GLM and the clinical manifestations (mass size, skin change, abscess, sinus and fistula formation): (1) self-limited stage, (2) congestive swelling stage, (3) abscess formation stage and (4) complex refractory stage. Treatment response was defined as partial response (improvement in all clinically significant symptoms, including pain, swelling, erythema, and induration) or complete response (complete resolution of the aforementioned symptoms) (Fig. 2) [85]. Notably, women do not have these discrete stages, they can be blurred and if they have multifocal disease then the different lesions can be at different stages and some skip stages.

### Self-limited stage

***Recommendation 27: GLM patients in the self-limited stage can be managed by watchful waiting using clinical and imaging surveillance. (Weak recommendation, low quality of evidence)***

Comments: One or more self-limited breast mass(es) usually present in the early stage of the GLM. The classic presentation is a breast mass less than 5 cm with mild pain and tenderness. The overlying skin can be normal and axillary lymphadenopathy are not common. No abscess is detected in physical examination or using an US. During this stage, GLM can be regarded as “white skin mass stage”. Watchful waiting by clinical examination and sonographic evaluation can be a reasonable approach at this stage. As a self-limited stage, symptoms may disappear or remain nonprogress for months or years. Only patients who show signs of disease progression need further treatment (Additional file 1: Fig. S2).

### Congestive swelling stage

***Recommendation 28: Patients with GLM in the hyperaemia and swelling progressive stage can be treated with oral corticosteroids, intralesional injection and topical corticosteroids, and non-corticosteroid immunosuppressive agents. (Strong recommendation, high quality of evidence)***

Comments: Hyperaemia and swelling progressive stage progresses from mass alone stage. The lesions are larger than or equal to 5 cm, with pain and without abscesses. The skin is hyperaemic and swollen, which can be regarded as “red skin mass stage”. Corticosteroids are considered as the front-line treatment option at this stage. Oral glucocorticoid can shrink the mass, reduce the pain and tenderness, and decrease inflammatory changes of the overlying skin. Subsequently, the dosage can be tapered off gradually over a period of 6–8 weeks. However, a small number of patients may experience relapse or recurrence of symptoms following tapering or discounting of the glucocorticoids is reduced or stopped. Then, GLM will rapidly enter into a refractory stage. For patients who cannot tolerate the adverse effects of corticosteroids therapy, wide local excision can be considered. Attention should be paid to the adverse effects of corticosteroids, such as Cushing’s syndrome and hirsutism, hypertension and increased glucose tolerance. Local treatment could be an option for those with mild inflammatory changes who are at higher risk for corticosteroid complications (Additional file 1: Fig. S3).

### Abscess formation stage

***Recommendation 29: Patients with GLM in the abscess formation stage can be performed with corticosteroids and wide local excision. (Strong recommendation, moderate quality of evidence)***

Comments: Abscess formation stage develops from mass alone stage or hyperaemia and swelling progressive stage. The formation of abscesses can be detected by physical examination or US. These lesions are large, often involving more than two quadrants, and are manifested in the acute stage, usually accompanied by axillary lymphadenopathy. Complicated sinuses and fistulas have not yet formed. Corticosteroid (oral, intralesional injection, topical), MTX and aspiration guided by US can be performed wide local excision can be performed if the aforementioned methods are not effective. For patients who refuse surgery, oral or topical corticosteroids can be applied if the single mass is limited, and intracavitary injection of triamcinolone acetonide guided by US is effective (Additional file 1: Fig. S4).

### Complex refractory stage

***Recommendation 30: As same as patients in the abscess formation stage, corticosteroids and wide local excision can also be applied for patients in the complex refractory stage. (Strong recommendation, moderate quality of evidence)***

Comments: In this advanced stage, lesions are extensively distributed to more than three quadrants or with abscesses, sinus, fistula and persistent wound infection (skin ulceration and pus). Patients in this stage need comprehensive treatment based on wide local excision [17]. Corticosteroid (oral, intralesional injection, topical), MTX and aspiration guided by US can be applied, and wide local excision should be performed if the aforementioned methods are not effective (Additional file 1: Fig. S5).

## Relapse and recurrence of GLM

Recurrence was considered as occurrence of inflammatory mass clinically or radiologically and was confirmed by Tru-cut biopsy [85]. In literature, recurrence rates of GLM were reported 15.4–24.8% [35, 86–89]. The patient who has a palpable mass at the time of discontinuation of medical therapy are at higher risk of relapse or recurrence than those without such finding. Younger age, corynebacterial infection, and pregnancy were associated with longer treatment durations [86]. Corynebacterial infection was associated with a 2.16–2.64 times higher risk of recurrence [73, 86]. Breast skin lesions were associated with a significantly higher odds of recurrence [35]. The difference of prolactin (PRL) level before and after treatment was an independent risk factor for recurrence and patients presenting with higher PRL after treatment than before treatment had a higher risk of recurrence [87].

## Future recommendations

GLM is a rare disease, and requires multi-center studies and meta-analysis for further understanding. We call for a comprehensive disease classification depending on clinical, radiological, and pathological criteria.

## Supplementary Information

**Additional file 1: Fig. S1** Histological staining of granulomatous lobular mastitis. **Fig. S2** Self-limited stage. **Fig. S3** Congestive swelling stage. **Fig. S4** Abscess formation stage. **Fig. S5** Complex refractory stage.

## Data Availability

The datasets used and/or analyzed during the current study are available from the corresponding author on reasonable request.

## Abbreviations

GLM: Granulomatous lobular mastitis
IGM: Idiopathic granulomatous mastitis
GM: Granulomatous mastitis
IGLM: Idiopathic granulomatous lobular mastitis
CNGM: Cystic neutrophilic granulomatous mastitis
CNB: Core needle biopsy
VAB: Vacuum-assisted biopsy
FNA: Fine needle aspiration
CCR: Complete clinical response
MTX: Methotrexate
PRL: Prolactin
