# Correction: Management of granulomatous lobular mastitis: an international multidisciplinary consensus (2021 edition)

**DOI:** 10.1186/s40779-022-00408-w

**Published:** 2022-08-23

**Authors:** Qian-Qian Yuan, Shu-Yuan Xiao, Omar Farouk, Yu-Tang Du, Fereshte Sheybani, Qing Ting Tan, Sami Akbulut, Kenan Cetin, Afsaneh Alikhassi, Rami Jalal Yaghan, Irmak Durur-Subasi, Fatih Altintoprak, Tae Ik Eom, Fatih Alper, Mustafa Hasbahceci, David Martínez-Ramos, Pelin Seher Oztekin, Ava Kwong, Cedric W. Pluguez-Turull, Kirstyn E. Brownson, Shirish Chandanwale, Mehran Habibi, Liu-Yi Lan, Rui Zhou, Xian-Tao Zeng, Jiao Bai, Jun-Wen Bai, Qiong-Rong Chen, Xing Chen, Xiao-Ming Zha, Wen-Jie Dai, Zhi-Jun Dai, Qin-Yu Feng, Qing-Jun Gao, Run-Fang Gao, Bao-San Han, Jin-Xuan Hou, Wei Hou, Hai-Ying Liao, Hong Luo, Zheng-Ren Liu, Jing-Hua Lu, Bin Luo, Xiao-Peng Ma, Jun Qian, Jian-Yong Qin, Wei Wei, Gang Wei, Li-Ying Xu, Hui-Chao Xue, Hua-Wei Yang, Wei-Ge Yang, Chao-Jie Zhang, Fan Zhang, Guan-Xin Zhang, Shao-Kun Zhang, Shu-Qun Zhang, Ye-Qiang Zhang, Yue-Peng Zhang, Sheng-Chu Zhang, Dai-Wei Zhao, Xiang-Min Zheng, Le-Wei Zheng, Gao-Ran Xu, Wen-Bo Zhou, Gao-Song Wu

**Affiliations:** 1 Department of Thyroid and Breast Surgery, Zhongnan Hospital of Wuhan University, Wuhan, 430071 China; 2 Department of Pathology, University of Chicago Pritzker School of Medicine, Chicago, IL 60637 USA; 3 Department of Surgical Oncology and Breast Surgery, Oncology Center, Faculty of Medicine, Mansoura University, Mansoura, 35516 Egypt; 4 Department of Breast Surgery, Beijing University of Chinese Medicine, Beijing, 100700 China; 5 Department of Infectious Diseases and Tropical Medicine, Faculty of Medicine, Mashhad University of Medical Sciences, Mashhad, 9177899191 Iran; 6 Breast Department, KK Women’s and Children’s Hospital, 100 Bukit Timah Road, Singapore, 229899 Singapore; 7 Department of Surgery, Department of Public Health, Department of Biostatistics, Bioinformatics and Medical Informatics, Inonu University Faculty of Medicine, 44280 Malatya, Turkey; 8 Department of General Surgery, Faculty of Medicine, Çanakkale Onsekiz Mart University, 17020 Çanakkale, Turkey; 9 Department of Radiology, Cancer Institute, Imam Khomeini Hospital, Tehran University of Medical Sciences, Tehran, 1419733141 Iran; 10 Department of Surgery, College of Medicine and Medical Sciences, Arabian Gulf University-Bahrain, Manama, 26671 Bahrain; 11 Department of Radiology, International Faculty of Medicine, Istanbul Medipol University, 34810 Istanbul, Turkey; 12 Department of General Surgery, Faculty of Medicine, Sakarya University, 54050 Sakarya, Turkey; 13 Department of Surgery, HiU Clinic, 170, Gwongwang-ro, Paldal-gu, Suwon, 16488 Korea; 14 Department of Radiology, Faculty of Medicine, Ataturk University, 25240 Erzurum, Turkey; 15 Academic Support and Education Center, Hırkai Serif District, Kececi Cesmesi Str, Doktorlar Building, B/7, 34091 Istanbul, Turkey; 16 Department of General and Digestive Surgery, Hospital General Castellon, Avda Benicassim S/N, 12812004 Castellón, Spain; 17 Radiology Department, Ankara Training and Research Hospital, 305018 Ankara, Turkey; 18 Department of Surgery, The University of Hong Kong, China; The University of Hong Kong-Shenzhen Hospital, Shenzhen, 518053 China; 19 University of Miami Health System and Miller School of Medicine, 1475 NW 12th Avenue, Miami, FL 33136 USA; 20 Department of Surgery, University of Utah, Huntsman Cancer Institute, Salt Lake City, UT 84112 USA; 21 Department of Pathology, Dr D Y Patil Medical College Hospital and Research Centre, Pimpri, Pune, 603203 India; 22 Department of Surgery, Johns Hopkins Breast Center at Bayview Campus, 4940 Eastern Avenue, Rm. A-562, Baltimore, MD 21224 USA; 23 Center for Evidence-Based and Translational Medicine, Zhongnan Hospital of Wuhan University, Wuhan, 430071 China; 24 Department of Diagnostic Ultrasound, Zhongnan Hospital of Wuhan University, Wuhan, 430071 China; 25 Department of Surgery, Affiliated Hospital of Inner Mongolia Medical University, Hohhot, 010110 China; 26 Center for Pathology and Molecular Diagnostics, Wuhan University, Wuhan, 430071 China; 27 Department of General Surgery, Fujian Provincial Hospital, Fuzhou, 350001 China; 28 The First Affiliated Hospital with Nanjing Medical University, Nanjing, 210029 China; 29 Key Laboratory of Hepatosplenic Surgery and the First Department of General Surgery, First Affiliated Hospital of Harbin Medical University, Harbin, 150007 China; 30 Department of Breast Surgery, Zhejiang University School of Medicine First Affiliated Hospital, Hangzhou, 310003 China; 31 Department of General Surgery, The Affiliated Hospital of Guizhou Medical University, Guiyang, 550004 China; 32 Department of General Surgery, Shanxi Provincial People’s Hospital, Taiyuan, 030012 China; 33 Department of Breast Surgery, Xinhua Hospital Affiliated to Shanghai Jiaotong University School of Medicine, Shanghai, 200092 China; 34 Department of Cardiothoracic Surgery, Zaoyang People’s Hospital, Zaoyang, 441299 Hubei China; 35 Department of Thyroid and Breast Surgery, The Second Hospital of Hebei Medical University, Shijiazhuang, 050004 China; 36 Department of General Surgery, Guangshan County People’s Hospital, Guangshan County, Xinxiang, 465499 Henan China; 37 Department of Breast Surgery, First Affiliated Hospital of Nanchang University, Nanchang, 330006 China; 38 Chinese Academy of Sciences, Beijing, 100045 China; 39 Department of General Surgery, School of Clinical Medicine, Tsinghua University, Beijing Tsinghua Changgung Hospital, Beijing, 102218 China; 40 Department of Breast and Thyroid Surgery, The First Affiliated Hospital of University of Science and Technology of China, Anhui Provincial Hospital, Hefei, 230001 China; 41 Department of Thyroid Surgery, First Affiliated Hospital of Kunming Medical University, Kunming, 650032 China; 42 Department of Oncology, Liwan Central Hospital of Guangzhou, Guangzhou, 510150 China; 43 Department of Breast Surgery, Peking University Shenzhen Hospital, Shenzhen, 518036 Guangdong China; 44 Department of Computed Tomography, Zhongnan Hospital of Wuhan University, Wuhan, 430071 China; 45 Department of General Surgery, Xinxiang Medical University First Affiliated Hospital, Xinxiang, 453100 Henan China; 46 Department of Breast Surgery, Guangxi Medical University Cancer Hospital, Nanning, 530021 China; 47 Department of General Surgery, Zhongshan Hospital Fudan University, Shanghai, 200032 China; 48 Department of Breast and Thyroid Surgery, Hunan Provincial People’s Hospital/The First Affiliated Hospital of Hunan Normal University, Changsha, 410005 China; 49 Department of Breast and Thyroid Surgery, Chongqing General Hospital, University of Chinese Academy of Sciences, Chongqing, 400013 China; 50 Department of General Surgery, Qinghai Province People’s Hospital, Xining, 810007 China; 51 Department of Thyroid and Breast Surgery, Qingdao Women and Children’s Hospital, Qingdao, 266000 Shandong China; 52 Department of Oncology, Xi’an Jiaotong University Second Affiliated Hospital, Xi’an, 710004 China; 53 Department of Cardiothoracic Surgery, Zaoyang First People’s Hospital, Zaoyang, 441299 Hubei China; 54 Department of Thyroid and Breast Surgery, Yichang Central People’s Hospital, Yichang, 443003 Hubei China; 55 Department of Thyroid Surgery, The Second Affiliated Hospital, Guizhou Medical University, Kaili, 556000 Guizhou China; 56 Department of General Surgery, Shanghai Changzheng Hospital, Shanghai, 200003 China; 57 Department of Surgery, Dongfeng General Hospital Affiliated with Hubei Medical College, Shiyan, 442001 Hubei China

## Correction: Military Medical Research (2022) 9:20 10.1186/s40779-022-00380-5

After publication of this article [1], it was brought to our attention that the second author’s name was incorrectly spelt. The correct name should be Shu-Yuan Xiao. The original publication has been corrected.
