# Supplementary material for: Management of granulomatous lobular mastitis: an international multidisciplinary consensus (2021 edition)
Source: Mil Med Res. 2022 Apr 26;9:20. doi: 10.1186/s40779-022-00380-5 (PMC9040252; doi:10.1186/s40779-022-00380-5)
Supplement: Supplementary file 1 — Additional file 1: Fig. S1 Histological staining of granulomatous lobular mastitis. Fig. S2 Self-limited stage. Fig. S3 Congestive swelling stage. Fig. S4 Abscess formation stage. Fig. S5 Complex refractory stage. [file 40779_2022_380_MOESM1_ESM.pdf]

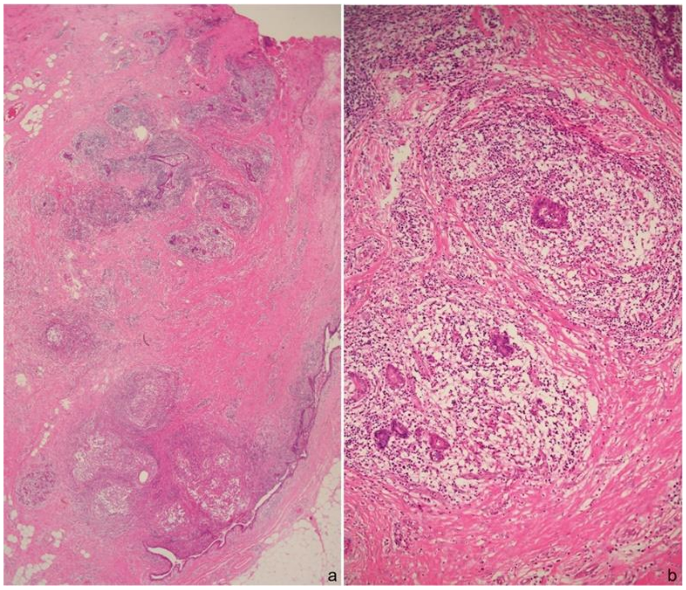

Fig. S1 Histological staining of granulomatous lobular mastitis. **a** hematoxylin-eosin stain (× 4). **b** hematoxylin-eosin stain (× 20)

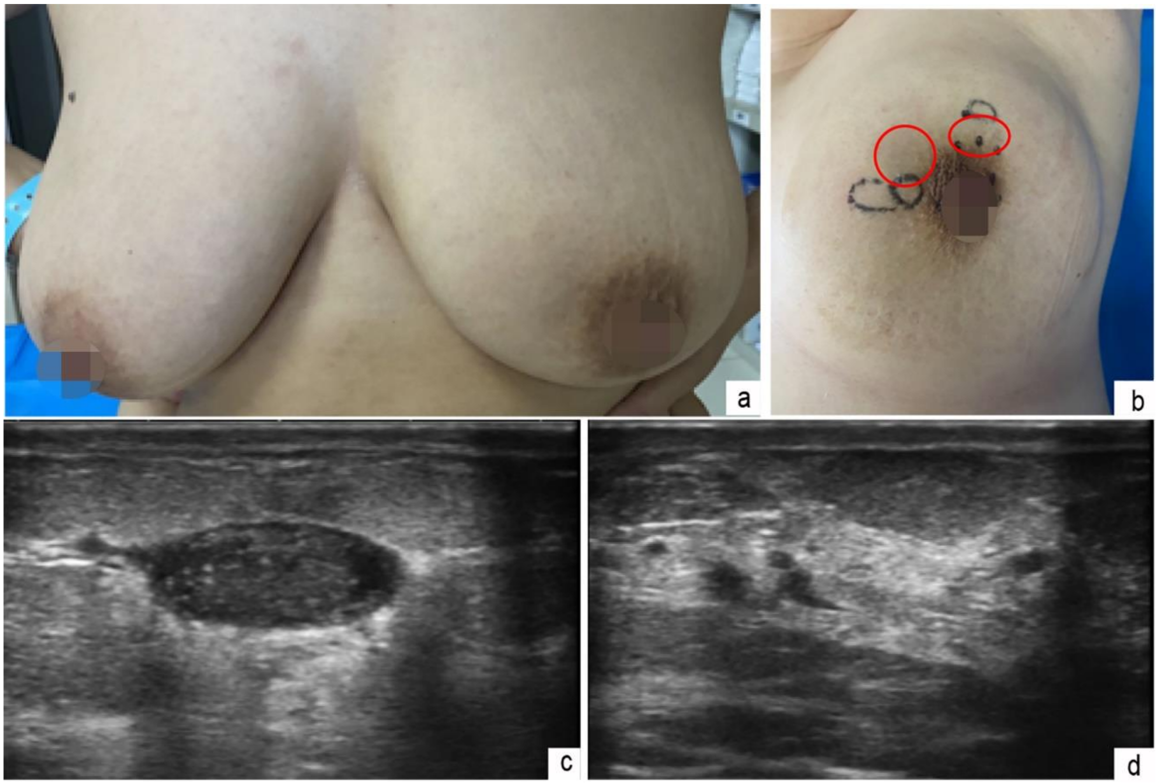

Fig. S2 Self-limited stage. **a, b** Granulomatous lobular mastitis in a 33-year-old woman with a painful mass of 6 weeks' duration in the upper inner quadrant of the left breast, without nipple retraction. **c, d** Hypoechoic masses were detected by ultrasound at the direction of 10 o'clock (3.5 cm × 0.9 cm) and 12 o'clock (0.5 cm × 0.4 cm, 0.3 cm × 0.2 cm, 0.4 cm × 0.2 cm)

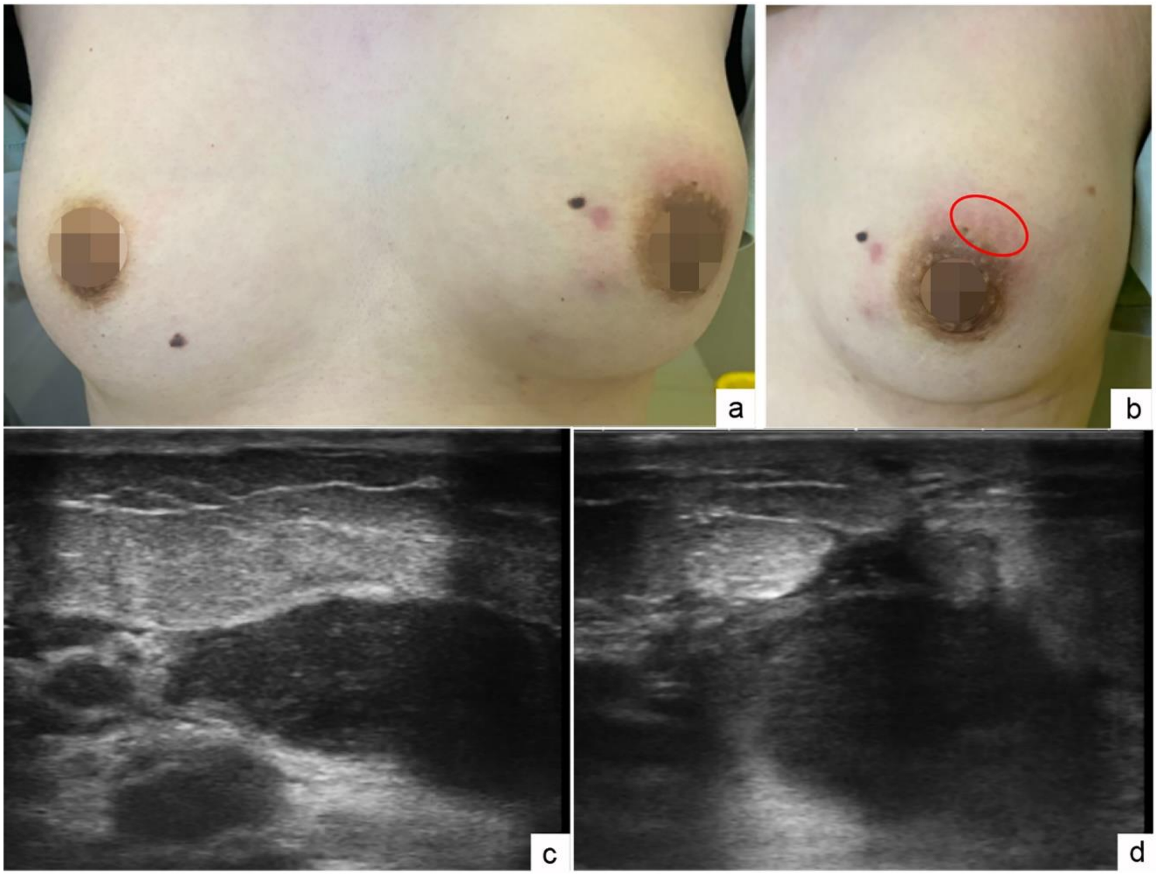

Fig. S3 Congestive swelling stage. **a, b** Granulomatous lobular mastitis in a 29-year-old woman with a painful mass of 3 months' duration in the upper outer quadrant of the left breast, with hyperaemia and swelling skin, without nipple retraction. **c, d** Larger hypoechoic mass (6.8 cm × 2.8 cm) was detected by ultrasound

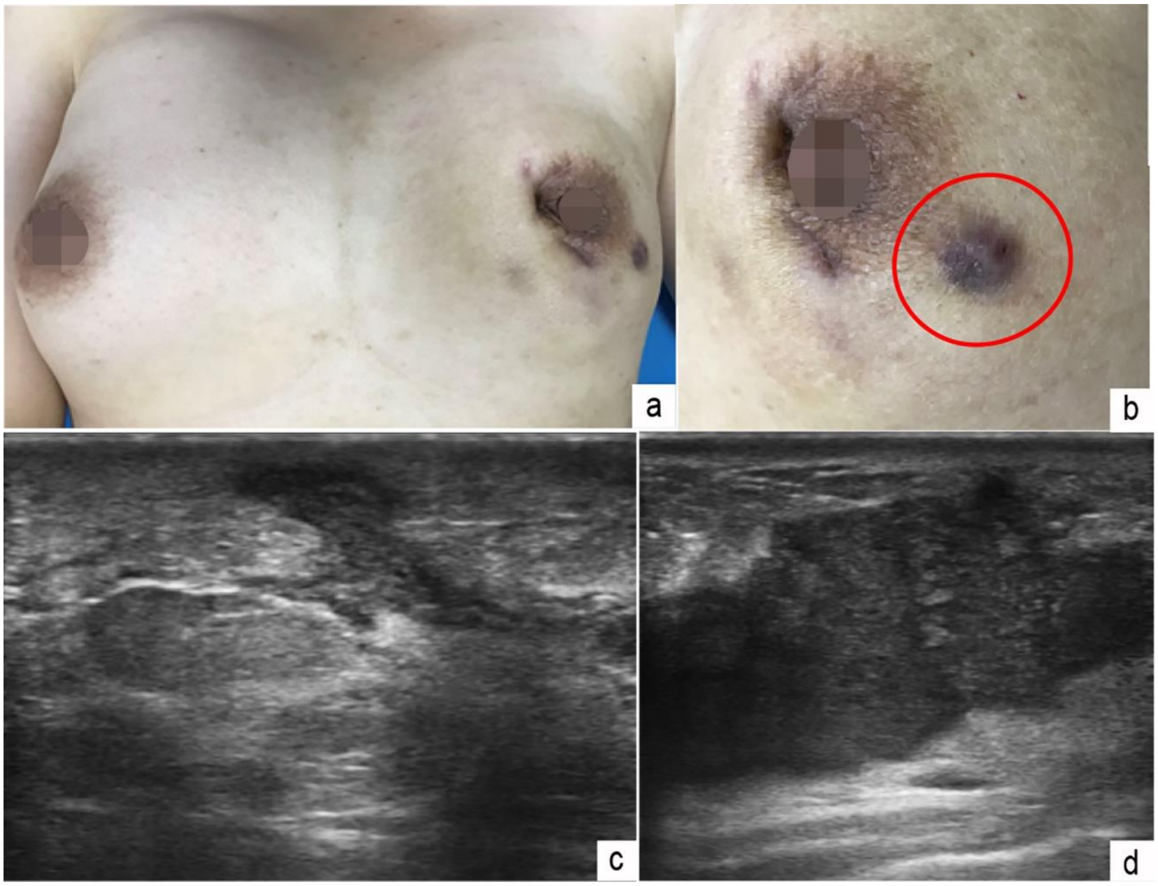

Fig. S4 Abscess formation stage. **a, b** Granulomatous lobular mastitis in a 35-year-old woman with a palpable mass of 4 weeks' duration in the outer lower quadrant of the left breast, with hyperaemia and swelling skin, without nipple retraction. **c, d** Irregular anechoic areas and abscesses (3.9 cm × 1.9 cm) were detected by ultrasound

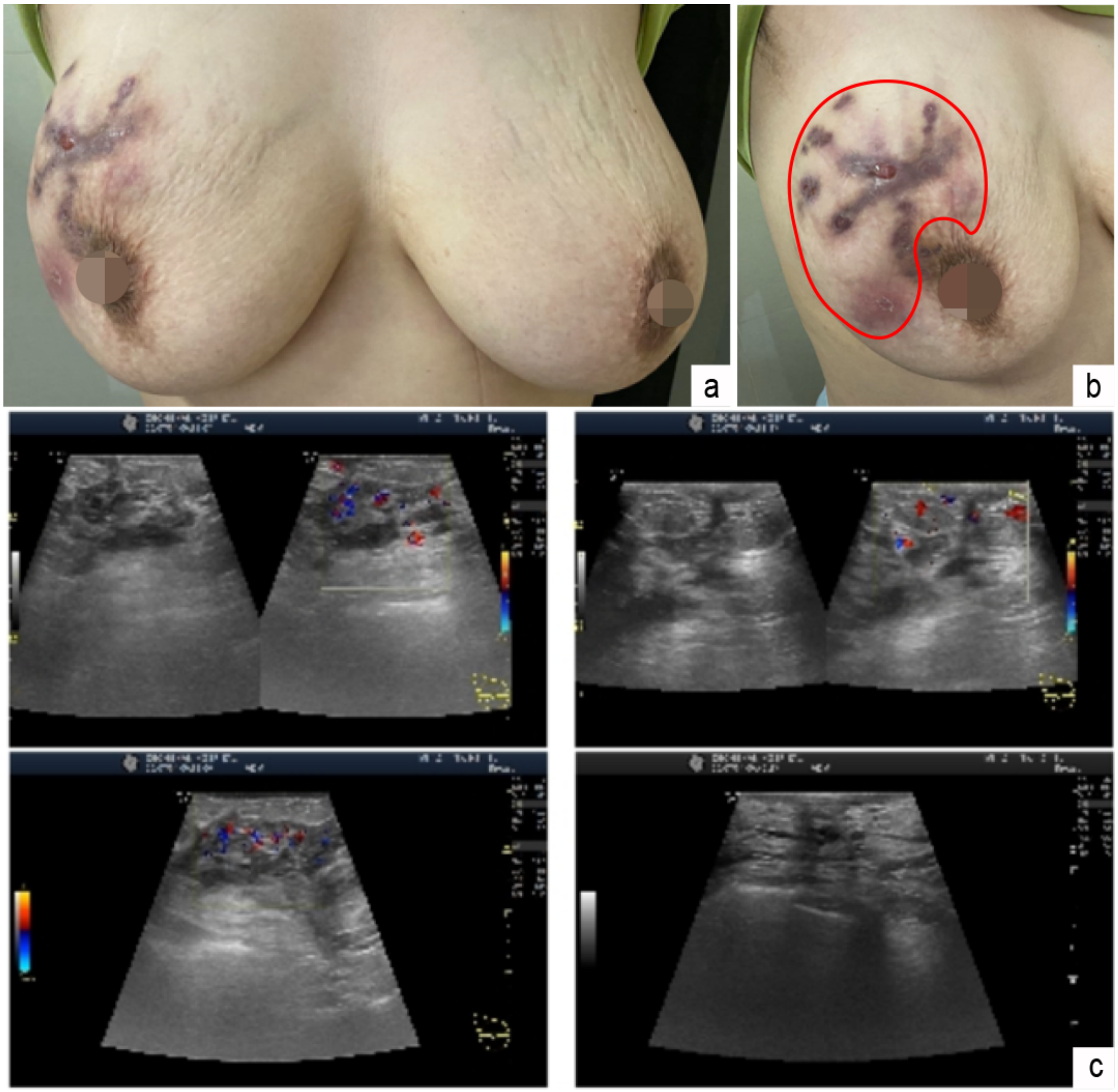

Fig. S5 Complex refractory stage. **a, b** Granulomatous lobular mastitis in a 33-year-old woman with a palpable mass and persistent wound infection of 6 months' duration in the outer upper and lower quadrants of the right breast, with hyperaemia and swelling skin, without nipple retraction. **c** Abscesses (5.3 cm × 0.6 cm), sinus, fistula, skin ulceration and pus were detected by ultrasound
